# Loss of XIAP facilitates switch to TNF*α*-induced necroptosis in mouse neutrophils

**DOI:** 10.1038/cddis.2016.311

**Published:** 2016-10-13

**Authors:** Simone Wicki, Ursina Gurzeler, W Wei-Lynn Wong, Philipp J Jost, Daniel Bachmann, Thomas Kaufmann

**Affiliations:** 1 Institute of Pharmacology, University of Bern, Bern, Switzerland; 2 Institute of Experimental Immunology, University of Zurich, Zurich, Switzerland; 3 III. Medizinische Klinik, Klinikum rechts der Isar, Technische Universität München, Munich, Germany

## Abstract

Neutrophils are essential players in the first-line defense against invading bacteria and fungi. Besides its antiapoptotic role, the inhibitor of apoptosis protein (IAP) family member X-linked IAP (XIAP) has been shown to regulate innate immune signaling. Whereas the role of XIAP in innate signaling pathways is derived mostly from work in macrophages and dendritic cells, it is not known if and how XIAP contributes to these pathways in neutrophils. Here we show that in response to bacterial lipopolysaccharides (LPS), mouse neutrophils secreted considerable amounts of tumor necrosis factor-*α* (TNF*α*) and interleukin-1*β* (IL-1*β*) and, in accordance with earlier reports, XIAP prevented LPS-induced hypersecretion of IL-1*β* also in neutrophils. Interestingly, and in contrast to macrophages or dendritic cells, *Xiap*-deficient neutrophils were insensitive to LPS-induced cell death. However, combined loss of function of XIAP and cIAP1/-2 resulted in rapid neutrophil cell death in response to LPS. This cell death occurred by classical apoptosis initiated by a TNF*α*- and RIPK1-dependent, but RIPK3- and MLKL-independent, pathway. Inhibition of caspases under the same experimental conditions caused a shift to RIPK3-dependent cell death. Accordingly, we demonstrate that treatment of neutrophils with high concentrations of TNF*α* induced apoptotic cell death, which was fully blockable by pancaspase inhibition in wild-type neutrophils. However, in the absence of XIAP, caspase inhibition resulted in a shift from apoptosis to RIPK3- and MLKL-dependent necroptosis. Loss of XIAP further sensitized granulocyte–macrophage colony-stimulating factor (GM-CSF)-primed neutrophils to TNF*α*-induced killing. These data suggest that XIAP antagonizes the switch from TNF*α*-induced apoptosis to necroptosis in mouse neutrophils. Moreover, our data may implicate an important role of neutrophils in the development of hyperinflammation and disease progression of patients diagnosed with X-linked lymphoproliferative syndrome type 2, which are deficient in XIAP.

Neutrophil granulocytes mature in the bone marrow and are released as terminally differentiated, short-lived cells into the blood where they constitute the most abundant leukocyte population in humans. Neutrophils are essential players in the host defense against bacterial and fungal infections. They are the first blood leukocytes to be recruited to sites of infection and play a central role in the first-line innate immune defense. Neutrophils express a large variety of surface receptors including innate immune receptors to recognize invading pathogens. Activation of those receptors leads to elimination of pathogens by phagocytosis, secretion of granule contents, reactive oxygen species and release of neutrophil extracellular traps.^1^ Toll-like receptor-4 (TLR4), a receptor for bacterial lipopolysaccharides (LPS), is one of the most important receptors constitutively expressed by neutrophils,^1, 2^ which upon activation enhances activity, cell survival and proinflammatory cytokine secretion.^2, 3^

Once activated at sites of inflammation, the lifespan of neutrophils has to be tightly controlled to avoid collateral damage to surrounding healthy tissue. Elimination of neutrophils is effectuated by apoptosis, whereas the exact mechanisms are not fully understood. Recently, it has been shown that neutrophils are eliminated by FAS/CD95 during viral and bacterial infections.^4^ However, neutrophils are also susceptible to other death receptors, in particular tumor necrosis factor receptor-1 (TNF-R1).^5, 6, 7^ Defects in the intrinsic (mitochondrial) apoptotic pathway prolong the lifespan of neutrophils.^8, 9^ Failure of neutrophils to undergo apoptosis contributes to chronic inflammation and tissue destruction and is found in various pathologies, such as rheumatoid arthritis, cystic fibrosis and chronic obstructive pulmonary disease.^10, 11, 12^

Members of the inhibitor of apoptosis protein (IAP) family are important modulators of cell death and cellular signaling.^13, 14^ X-linked IAP (XIAP) has first been recognized for its antiapoptotic function and it remains the only family member that can directly block the catalytic activities of caspase-3/-7 and, to lesser extent, caspase-9, via its baculoviral IAP repeat (BIR) domains.^13, 15^ XIAP is a critical discriminator between FAS/CD95-induced type I and type II apoptotic signaling.^16, 17^ In contrast to FAS/CD95, TNF-R1 does not typically induce cell death at physiological concentrations in most cell types, but rather triggers strong activation of NF-*κ*B and subsequent transcription of proinflammatory and prosurvival genes. Depending on the cellular context, however, TNF-R1 signaling can switch from NF-*κ*B activation to apoptosis or necroptosis, respectively.^18, 19, 20^ Ubiquitylation of RIPK1 favors NF-*κ*B activation and in turn survival, whereas deubiquitylation of RIPK1 proceeds to complex II formation, which results either in caspase-mediated apoptosis or RIPK3- and MLKL-mediated necroptosis.^19, 21, 22, 23^ In contrast to cIAP1/-2, which act as E3 ubiquitin ligases within proximal signaling complexes of death receptors and certain TLRs (TLR2 and TLR4), XIAP has not been directly implicated in TNF-R1 signaling and has never been found within proximal signaling complexes I and II.^14, 24, 25, 26^ However, a role for XIAP in TNF*α*-induced cell death, likely outside of complex I, has recently been shown in macrophages and dendritic cells (DCs).^27, 28^

Similar to cIAP1/-2, XIAP contains a RING domain that functions as E3 ubiquitin ligase regulating NF-*κ*B and MAPK signaling pathways.^14, 29, 30^ The best example currently is the positive regulation of NOD1/2-dependent signaling by XIAP upon detection of intracellular bacteria.^31, 32, 33^ A crucial physiological role of XIAP in immunity is supported by loss-of-function mutations in patients suffering from the primary immunodeficiency disorder X-linked lymphoproliferative syndrome 2 (XLP2).^34^

The role of XIAP in innate immune signaling is almost exclusively derived from work in macrophages and DCs.^27, 28, 35, 36^ To date, little is known about the function and importance of XIAP in neutrophils, despite their prominence in the blood and at sites of infection.^11^ Neutrophils are implicated in several pathologies in which XIAP might have a role, including rheumatoid arthritis or inflammatory bowel disease.^27, 37, 38, 39^ Aberrant expression of XIAP (as well as cIAP1/-2) has been reported in a variety of human cancers and is associated with poor prognosis, chemoresistance and disease progression.^40^ Specific targeting of individual IAPs using small-molecule inhibitors called Smac (second mitochondria-derived activator of caspase) mimetics (or IAP inhibitors) constitutes a potential promising novel therapeutic approach.^41^

Here, we determined the role of XIAP in response to LPS and subsequent TNF*α*>TNF-R1 signaling in mouse neutrophils. We observed that XIAP largely maintains viability of LPS-stimulated neutrophils when cIAP1/-2 are inhibited. However, additional loss of XIAP resulted in rapid cell death via TNF*α*- and RIPK1-dependent apoptosis. We show that mouse neutrophils were intrinsically sensitive to caspase-dependent apoptosis in response to high TNF*α* and that a switch to necroptosis did not occur when caspases were blocked, despite high expression levels of RIPK1, RIPK3 and MLKL. However, loss of XIAP promoted a shift towards necroptosis. These results implicate a previously unrecognized direct regulatory role of XIAP downstream of TNF-R1 in neutrophils.

## Results

### LPS induces production of TNF*α* in mouse neutrophils and exacerbated IL-1*β* release upon loss of XIAP

**Figure 1:**
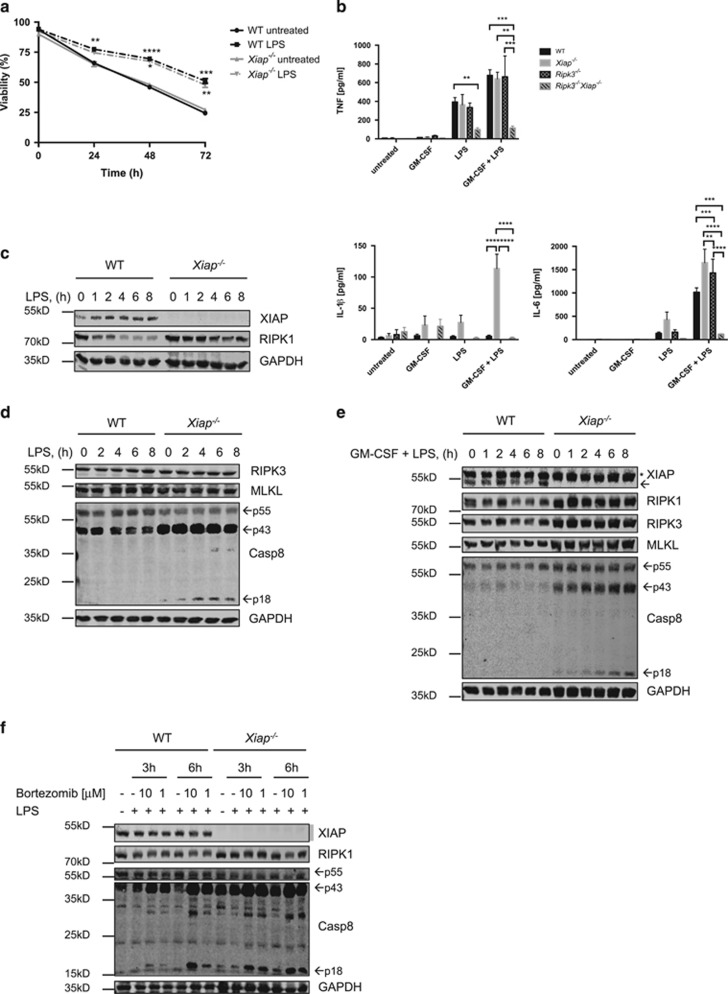
Loss of XIAP results in excessive secretion of IL-1*β* in the absence of increased cell death and stabilization of RIPK1. (**a**) Assessment of viability in WT and *Xiap*^−*/*−^ neutrophils upon treatment with LPS (100 ng/ml) for indicated time points; *n*≥6, mean±S.E.M. (**b**) WT, *Xiap*^−*/*−^, *Ripk3*^−*/*−^ and *Ripk3*^−*/*−^*Xiap*^−*/*−^ neutrophils were treated with LPS (100 ng/ml) for 24 h, and supernatants were assessed for TNF*α*, IL-6 and IL-1*β* by ELISA; *n*≥3, mean±S.E.M. (**c** and **d**) Unprimed and (**e**) GM-CSF-primed (1 ng/ml for 30 min) WT and *Xiap*^−*/*−^ neutrophils were stimulated with LPS (100 ng/ml) for 0–8 h, and lysates were subjected to quantitative immunoblot using near-infrared fluorescence. Presented immunoblots are representative of at least two independent experiments. *Nonspecific band. (**f**) WT and *Xiap*^−*/*−^ neutrophils were pre-treated for 30 min with bortezomib (1 and 10 *μ*M), and stimulated with LPS (100 ng/ml) for 3 and 6 h. Lysates were assessed by immunoblot. Presented immunoblots are representative of at least two independent experiments. All experiments were performed with *in vitro* differentiated neutrophils. *P<0.05, **P<0.01, ***P<0.005, ****P<0.001

We first assessed viabilities over time and cytokine production of wild-type (WT) and *Xiap*-deficient (henceforth termed *Xiap*^−/−^) neutrophils stimulated with ultrapure LPS. Besides primary neutrophils isolated from bone marrow, which can only be obtained in limited numbers, neutrophils were differentiated *in vitro* using conditional Hoxb8, which is a suitable tool for the generation of large quantities of functional mouse neutrophils.^42, 43, 44, 45^ LPS did not increase cell death in primary or *in vitro* differentiated WT neutrophils but induced the release of TNF*α* and IL-6, which was further enhanced upon priming with GM-CSF (Figures 1a, b and Supplementary Figures S1a–d). LPS induced comparable TNF*α* and IL-6 levels in *Xiap*^−/−^ neutrophils, but, interestingly, loss of XIAP did not increase cell death, in contrast to previous reports on macrophages and DCs.^27, 28^ It has been shown for macrophages and DCs that in the absence of XIAP, or alternatively cIAP1/-2, LPS stimulation alone causes exacerbated IL-1*β* secretion.^28, 35^ Consistent with these findings, GM-CSF priming followed by LPS stimulation promoted excessive IL-1*β* secretion in *Xiap*^−/−^, but not in WT, neutrophils that was preceded by proteolytic processing of procaspase-8 (Figures 1b, d, e and Supplementary Figures S1b–d and f). However, LPS stimulation induced comparable levels of pro-IL-1*β* and NLRP3 in both genotypes, and this was further enhanced by GM-CSF (Supplementary Figure S1e). IL-1*β* secretion was abrogated upon additional loss of *Ripk3* (Figure 1b). Furthermore, additional loss of caspase-1/-11 in *Xiap*^−/−^ neutrophils partially reduced but did not completely abolish IL-1*β* secretion (Supplementary Figure S1b), which is consistent with findings in DCs.^28^ Interestingly, LPS induced a rapid decrease in RIPK1 in WT neutrophils, which was less prominent in *Xiap*^−/−^ neutrophils (Figures 1c and e). This decrease of RIPK1 was not prevented upon neutralizing TNF*α* or blocking apoptotic caspases (Supplementary Figure S1g). Immunoblot analysis showed that both unprimed and primed mouse neutrophils express readily detectable levels of RIPK1, RIPK3 and MLKL, all of which are necessary for a cell to undergo necroptotic cell death (Figures 1c–e). Proteasomal inhibition using bortezomib induced a mobility shift but did not restore RIPK1 protein levels (Figure 1f). Taken together, LPS-stimulated *Xiap*^−/−^ neutrophils secrete comparable levels of TNF*α* compared with WT cells, but massively increased levels of IL-1*β* upon priming. Furthermore, XIAP seems to regulate the stability of RIPK1 in response to LPS.

### LPS kills cIAP1/-2-depleted neutrophils in a TNF*α*-dependent manner only upon additional loss of XIAP

**Figure 2:**
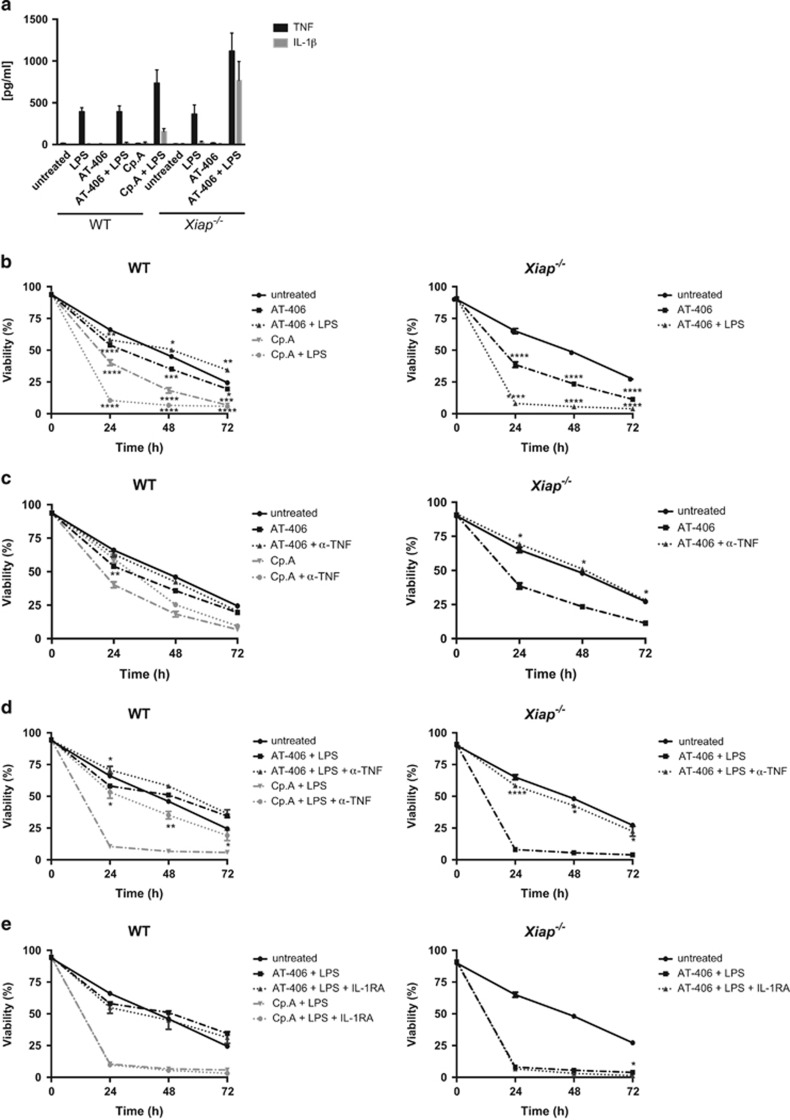
LPS kills cIAP1/-2-depleted neutrophils in a TNF*α*-dependent manner only upon additional loss of XIAP. (**a**) Supernatants were collected from WT and *Xiap*^−*/*−^ neutrophils pre-treated with AT-406 (1 *μ*M) or Cp.A (500 nM) for 30 min as indicated and stimulated with LPS for 24 h. TNF*α* and IL-1*β* in the supernatants were measured by ELISA; *n*≥3, mean±S.E.M. (**b**) Assessment of viability of WT and *Xiap*^−*/*−^ neutrophils pre-treated with AT-406 (1 *μ*M) or Cp.A (500 nM) for 30 min and subsequently stimulated with LPS (100 ng/ml) for indicated time points; *n*≥6, mean±S.E.M. (**c** and **d**) WT and *Xiap*^−*/*−^ neutrophils were pre-treated with TNF antagonist (10 *μ*g/ml) for 30 min, incubated with (**c**) AT-406 (1 *μ*M) or Cp.A (500 nM) and stimulated with (**d**) LPS (100 ng/ml) for indicated time points. Viability was assessed by flow cytometry; *n*≥3, mean±S.E.M. (**e**) WT and *Xiap*^−*/*−^ neutrophils were pre-treated with IL-1R antagonist (10 *μ*g/ml) for 30 min, further incubated with AT-406 (1 *μ*M) or Cp.A (500 nM) and stimulated with LPS (100 ng/ml) for indicated time points. Viability was assessed by flow cytometry; *n*≥3, mean±S.E.M. All experiments were performed with *in vitro* differentiated neutrophils. Same data sets of untreated control and Smac mimetics (SM)±LPS are shown in the different subpanels. *P<0.05, **P<0.01, ***P<0.005, ****P<0.001

To assess the involvement of XIAP in the regulation of TLR4 signaling in neutrophils, we used the monovalent Smac mimetic AT-406, which, despite its ability to bind to all three IAPs, predominantly antagonizes cIAP1/-2 (cIAP1, *K*_i_=1.9 nM; cIAP2, *K*_i_=5.1 nM; XIAP, *K*_i_=66.4 nM),^46^ and the bivalent Smac mimetic compound A (Cp.A) with high binding affinity to XIAP and cIAP1/-2.^47^ In contrast to macrophages,^48^ treatment of WT and *Xiap*^−*/*−^ neutrophils with Smac mimetics alone did not elicit any detectable secretion of TNF*α* or IL-1*β* (Figure 2a and Supplementary Figure S2a). Whereas AT-406 did not induce cell death in WT neutrophils, a small but significant increase in cell death was observed in *Xiap*^−*/*−^ neutrophils as well as in Cp.A-treated WT neutrophils (Figure 2b). This cell death could be blocked by TNF*α* neutralization (Enbrel) (Figure 2c). Importantly, stimulation with LPS had a massive negative impact on viability when the function of all IAPs was lost. Whereas AT-406-treated WT neutrophils were refractory to killing by LPS, the same treatment in *Xiap*^−*/*−^ neutrophils induced complete cell death within 24 h, as did Cp.A plus LPS in WT neutrophils (Figure 2b and Supplementary Figure S2b). To test the contribution of TNF*α* or IL-1*β* to the observed cell death, we pre-treated the cells with Enbrel or an IL-1*β* receptor antagonist (Anakinra). As shown in (Figures 2d, e and Supplementary Figure S2c), blocking of IL-1R had no impact on cell viability, whereas antagonism of TNF*α* almost completely abolished cell death. Taken together, only loss of all three IAP sensitizes neutrophils to LPS-induced killing, which depends on TNF*α* but is independent of IL-1*β*. Furthermore, the data show that XIAP crucially controls survival of LPS-stimulated neutrophils, as it fully maintains viability when cIAP1/-2 are functionally lost.

### LPS induces RIPK1-dependent apoptosis in IAP-depleted neutrophils

**Figure 3:**
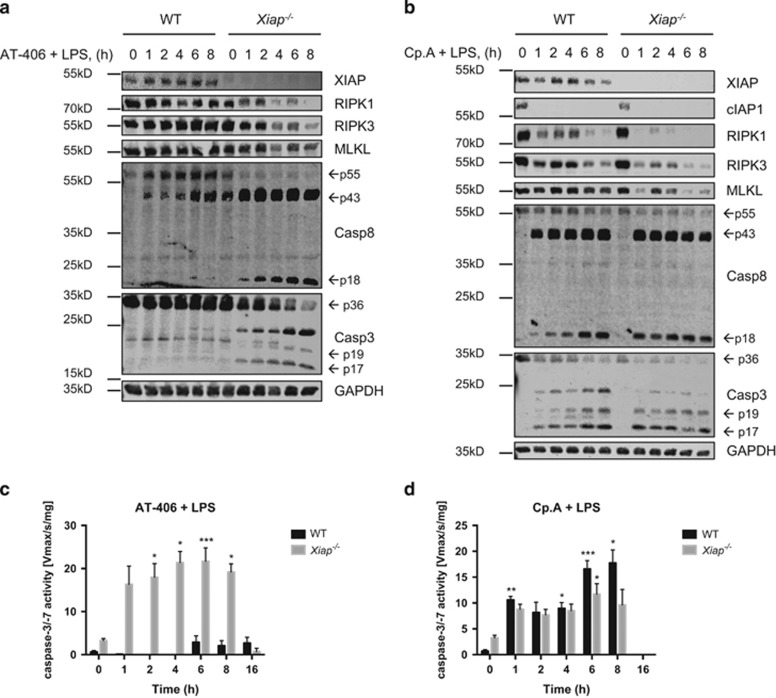
Combined treatment with LPS and Smac mimetics activates apoptotic caspases. (**a** and **b**) WT and *Xiap*^−*/*−^ neutrophils were pre-treated with (**a**) AT-406 (1 *μ*M) or (**b**) Cp.A (500 nM) for 30 min followed by treatment with LPS (100 ng/ml) for indicated time points. Lysates were assayed by immunoblot. Presented immunoblots are representative of at least two independent experiments. (**c** and **d**) WT and *Xiap*^−*/*−^ neutrophils were pre-treated with either (**c**) AT-406 (1 *μ*M) or (**d**) Cp.A (500 nM) for 30 min and subsequently incubated with LPS (100 ng/ml) for 0–16 h. Lysates are assayed for caspase-3/-7 activity by fluorogenic DEVDase assay; *n*≥3, mean±S.E.M. All experiments were performed with *in vitro* differentiated neutrophils. *P<0.05, **P<0.01, ***P<0.005

**Figure 4:**
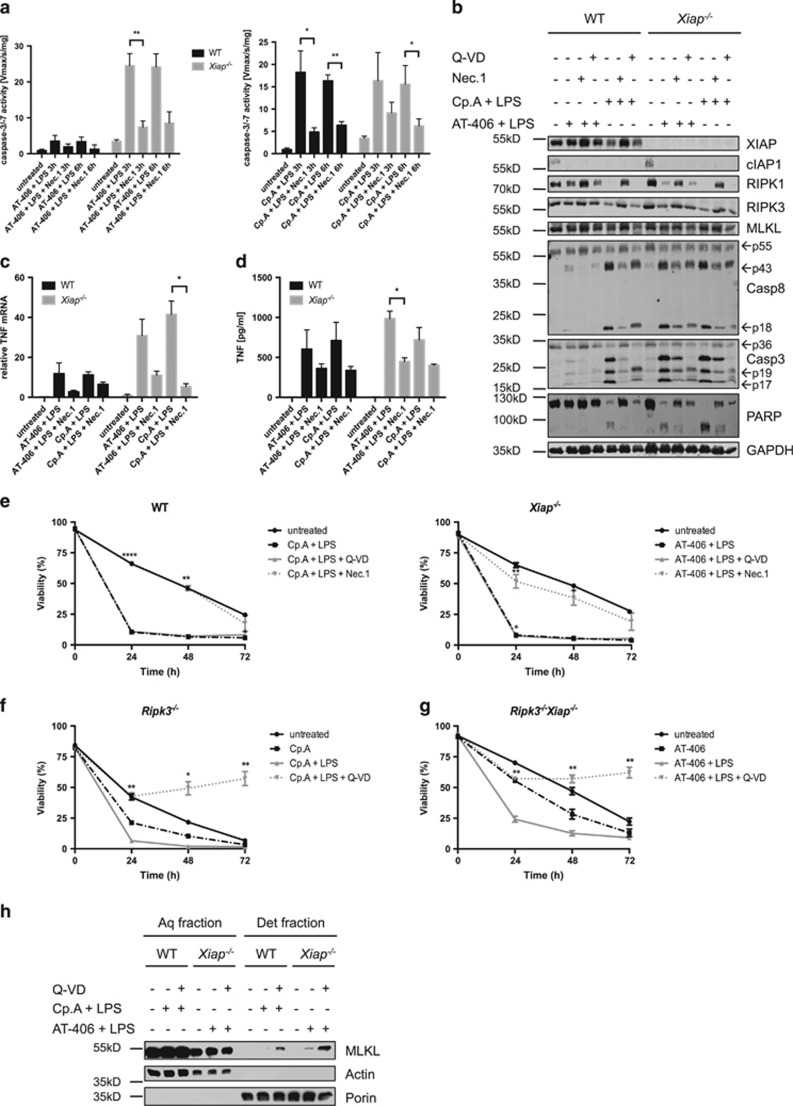
LPS plus Smac mimetics induces RIPK1-dependent apoptosis that switches to necroptosis upon caspase inhibition. (**a**) WT and *Xiap*^−*/*−^ neutrophils were pre-treated with Nec.1 (20 *μ*M) for 30 min, AT-406 (1 *μ*M) or Cp.A (500 nM) for 30 min and subsequently incubated with LPS (100 ng/ml) for 3 and 6 h. Lysates were assayed for caspase-3/-7 activity; *n*≥4, mean±S.E.M. (**b**) WT and *Xiap*^−*/*−^ neutrophils were pre-treated with Nec.1 (20 *μ*M, 30 min) or Q-VD-OPh (20 *μ*M, 30 min), then with either AT-406 (1 *μ*M) or Cp.A (500 nM), and incubated with LPS (100 ng/ml) for 6 h. Lysates were assayed by immunoblot. Presented immunoblots are representative of at least two independent experiments. (**c**) qPCR analysis of *Tnfα* in WT and *Xiap*^−*/*−^ neutrophils pre-treated with Nec.1 (20 *μ*M, 30 min) followed by either AT-406 (1 *μ*M) or Cp.A (500 nM), for another 30 min and stimulated with LPS (100 ng/ml) for 4 h. *Hprt* was used as reference gene; *n*=3, mean±S.E.M. (**d**) WT and *Xiap*^−*/*−^ neutrophils were preincubated with Nec.1 (20 *μ*M, 30 min), treated with either AT-406 (1 *μ*M) or Cp.A (500 nM) and stimulated with LPS (100 ng/ml) for 6 h. Supernatants were analyzed for TNF*α* by ELISA; *n*≥4, mean±S.E.M. (**e**) WT and *Xiap*^−*/*−^ neutrophils were pre-treated with Q-VD-OPh (20 *μ*M) or Nec.1 (20 *μ*M) for 30 min, treated with AT-406 (1 *μ*M) or Cp.A (500 nM) for 30 min and subsequently stimulated with LPS (100 ng/ml) for different time points. Viability was determined by flow cytometry; *n*≥3, mean±S.E.M. Data sets of untreated control from Figure 1a and SM+LPS from Figure 2b are included. (**f**) *Ripk3*^−*/*−^ and (**g**) *Ripk3*^−*/*−^*Xiap*^−*/*−^ neutrophils were pre-treated with Q-VD-OPh (20 *μ*M) for 30 min, incubated with Cp.A (500 nM) or AT-406 (1 *μ*M), respectively, and stimulated with LPS (100 ng/ml) for indicated time points. Viability was assessed by flow cytometry; *n*≥3, mean±S.E.M. (**h**) WT and *Xiap*^−*/*−^ neutrophils were pre-treated with Q-VD (20 *μ*M) for 30 min followed by administration of Cp.A (500 nM) or AT-406 (1 *μ*M), respectively, for 30 min and stimulated with LPS for 6 h. Fractionation by phase separation was performed and lysates were assayed by quantitative immunoblot. Presented immunoblots are representative of at least two independent experiments. All experiments were performed with *in vitro* differentiated neutrophils. *P<0.05, **P<0.01, ***P<0.005, ****P<0.001

To investigate the type of TNF*α*-dependent cell death induced by LPS plus Smac mimetics (Figure 2), we analyzed proteolytic processing of apoptotic caspases in WT and *Xiap*^−*/*−^ neutrophils by immunoblotting. Cotreatment of AT-406 with LPS induced rapid and strong proteolytic cleavage of procaspase-8 and -3 into their putatively active forms in *Xiap*^−*/*−^ but, importantly, not in WT neutrophils (Figure 3a). Coaddition of LPS and Cp.A also led to a strong cleavage of caspases; however, this was now comparable between the two genotypes (Figure 3b). Furthermore, caspase-8 processing correlated with a decrease of its substrates RIPK1 and RIPK3. Cleavage of effector caspase substrate PARP concurred with the processing of procaspase-3, suggesting that this caspase was indeed active (Figure 4b). Enzymatic activation of caspases-3/-7 was confirmed by fluorogenic (DEVDase) assay. Upon treatment of *Xiap*^−*/*−^ neutrophils with AT-406 plus LPS, or WT or *Xiap*^−*/*−^ neutrophils with Cp.A plus LPS, caspase-3/-7 was activated within 1 h and peaked after 6–8 h (Figures 3c and d). Importantly, caspase-3/-7 activation was dependent on the enzymatic activity of RIPK1, as its specific inhibitor necrostatin-1 (Nec.1) blocked DEVDase activity (Figure 4a), processing of procaspases and the decrease of RIPK1 and RIPK3 (Figure 4b). Nec.1 consequently inhibited LPS-induced cell death in IAP-depleted neutrophils (Figure 4e). In accordance with our previous observation that the observed cell death was TNF*α* dependent, necrostatin-1 blocked LPS-induced *de novo* production and release of TNF*α* (Figures 4c and d).

Interestingly, whereas Nec.1 was highly efficient in preventing LPS plus Smac mimetics-induced cell death in WT or *Xiap*^−*/*−^ neutrophils, addition of the pancaspase inhibitor Q-VD-OPh had no protective effect (Figure 4e). Genetic loss of *Ripk3* did not prevent cell death by LPS plus Smac mimetics. However, on a *Ripk3*^−*/*−^ genetic background, pancaspase inhibition now fully protected the cells under these experimental conditions (Figures 4f and g). When IAP-depleted neutrophils were stimulated with LPS, Q-VD-OPh induced the translocation of the RIPK3 substrate and critical effector molecule of necroptosis, MLKL, from a soluble (aqueous) fraction to an integral membrane-enriched (detergent) fraction (Figure 4h). Collectively, these data indicate that XIAP is crucial in maintaining neutrophil viability in response to LPS and that in the absence of all three IAPs, rapid caspase-dependent apoptosis is initiated, which depends on RIPK1 activity and requires autocrine TNF*α* but is independent of RIPK3. Blocking of caspases may then shift the cell death from apoptosis to RIPK3- and MLKL-dependent necroptosis.

### XIAP blocks the switch from TNF*α*-induced apoptosis to necroptosis in mouse neutrophils

**Figure 5:**
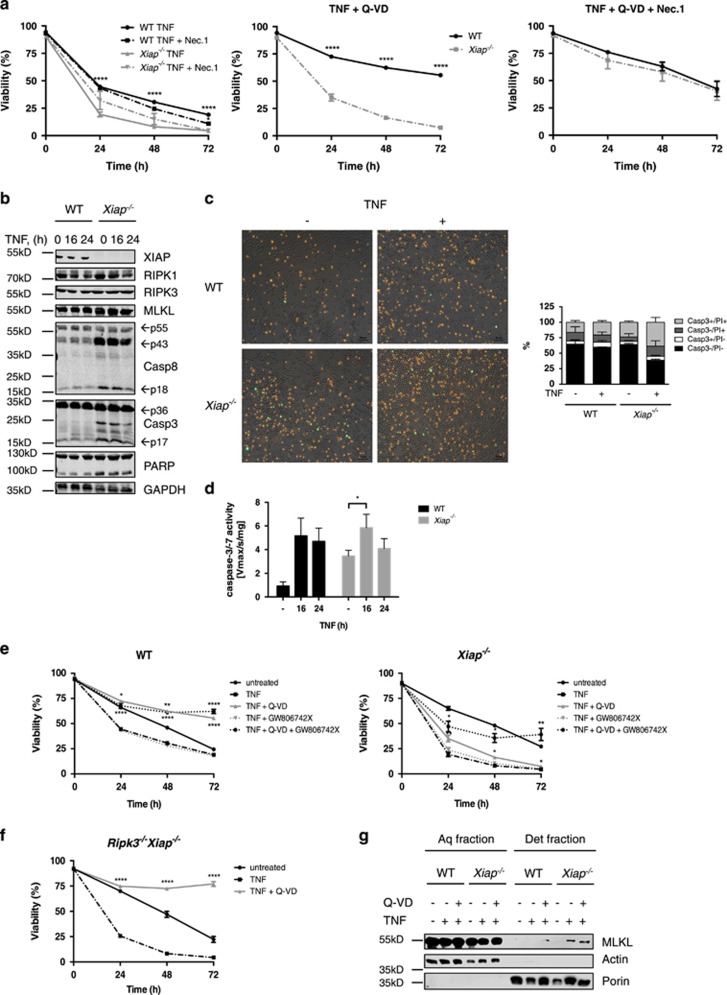
XIAP prevents Q-VD-OPh induced shift from apoptosis to necroptosis in response to high concentrations of TNF*α* in neutrophils. (**a** and **e**) WT and *Xiap*^−*/*−^ neutrophils were pre-treated with Q-VD-OPh (20 *μ*M) and (**a**) Nec.1 (20 *μ*M) or (**e**) the MLKL inhibitor GW806742X (1 *μ*M) for 30 min and subsequently stimulated with recombinant TNF*α* (100 ng/ml) for indicated time points. Viability was assessed by flow cytometry; *n*≥4, mean±S.E.M. Same data sets of TNF±Q-VD treatments are shown in (**a** and **e**); data sets of untreated control from Figure 1a are included in (**e**). (**b**) WT and *Xiap*^−*/*−^ neutrophils were stimulated with TNF*α* (100 ng/ml) for 16 and 24 h. Lysates were assayed by immunoblot. Presented immunoblots are representative of at least two independent experiments. (**c**) WT and *Xiap*^−*/*−^ neutrophils were treated with TNF*α* (100 ng/ml) for 24 h. Cells were stained for active caspase-3/-7 (green) using CellEvent Caspase-3/-7 Green Detection Reagent and PI (red). Presented images are representative of at least two independent experiments. Additionally, stained cells were analyzed by flow cytometry. (**d**) WT and *Xiap*^−*/*−^ neutrophils were treated with TNF*α* (100 ng/ml) for 16 and 24 h. Lysates were assayed for caspase-3/-7 activity using fluorogenic DEVDase assay; *n*≥3, mean±S.E.M. (**f**) *Ripk3*^−*/*−^*Xiap*^−*/*−^ neutrophils were preincubated with Q-VD-OPh (20 *μ*M) and further stimulated with TNF*α* (100 ng/ml) for indicated time points. Viability was assessed by flow cytometry; *n*≥6, mean±S.E.M. Data set of untreated control from Figure 4g is included to facilitate comparison. (**g**) WT and *Xiap*^−*/*−^ neutrophils were pre-treated with Q-VD-OPh (20 *μ*M) for 30 min followed by administration of TNF*α* (100 ng/ml) for 16 h. Fractionation by phase separation was performed and lysates were assayed by quantitative immunoblot. Presented immunoblots are representative of at least two independent experiments. All experiments were performed with *in vitro* differentiated neutrophils. *P<0.05, **P<0.01, ***P<0.005, ****P<0.001

Based on the obtained importance of TNF*α* in LPS plus Smac mimetics-induced neutrophil cell death, we next studied the role of XIAP downstream of TNF-R1. As reported previously, low concentrations of TNF*α* promote survival, whereas high doses induce apoptosis in neutrophils. Consistent with previous reports,^5, 7^ both primary and *in vitro* differentiated WT and *Xiap*^−*/*−^ neutrophils underwent significantly increased cell death in response to high concentrations of TNF*α* (Figure 5a and Supplementary Figure S3a). Furthermore, sensitivity to lower concentrations (10 and 1 ng/ml, respectively) of TNF*α* was strongly increased upon treatment with Smac mimetics (Supplementary Figures S3c and d). TNF*α*-induced cell death occurred by apoptosis, as assessed by the processing of caspase-8, activation of caspase-3 and PARP cleavage (Figures 5b–d and Supplementary Figure S3b). Importantly, pancaspase inhibition could fully rescue TNF*α*-induced cell death in WT neutrophils but had only minimal protective effects in *Xiap*^−*/*−^ neutrophils (Figure 5a and Supplementary Figure S3a). Complete protection in *Xiap*^−*/*−^ neutrophils could only be achieved upon combined inhibition of caspases and RIPK1 or MLKL, respectively (Figures 5a, e and Supplementary Figure S3a). These data indicate that while caspase inhibition fails to convert TNF*α*-induced apoptosis to necroptosis in WT neutrophils, such a switch does occur upon loss of XIAP. In support of this model, *Ripk3*^−*/*−^*Xiap*^−*/*−^ neutrophils were equally sensitive as *Xiap*^−*/*−^ neutrophils to TNF*α*-induced killing, but this cell death was now fully blockable by Q-VD-OPh (Figure 5f). Furthermore, increased amounts of MLKL translocated to an integral membrane-enriched fraction in TNF*α* plus Q-VD-OPh-stimulated *Xiap*^−*/*−^ neutrophils (Figure 5g). Taken together, our data show that high concentrations of TNF*α* elicit apoptosis in both WT and *Xiap*^−*/*−^ neutrophils. However, a switch to necroptosis upon inhibition of caspases does not occur in WT neutrophils but is enabled when XIAP is lost.

### Loss of XIAP sensitizes GM-CSF-primed neutrophils towards TNF*α*-induced cell death

**Figure 6:**
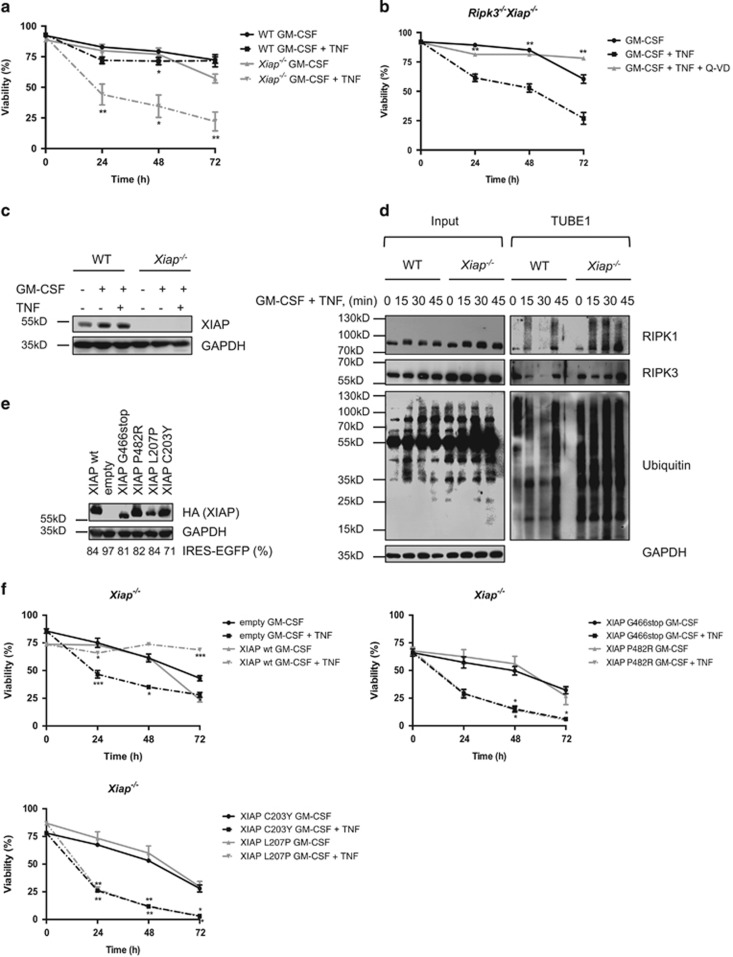
XIAP confers resistance to TNF*α*-induced cell death in GM-CSF-primed neutrophils. (**a**) WT and *Xiap*^−*/*−^ neutrophils were primed with GM-CSF (1 ng/ml) for 30 min before stimulation with TNF*α* (100 ng/ml) for indicated time points. Viability was assessed by flow cytometry; *n*≥4, mean±S.E.M. (**b**) GM-CSF-primed *Ripk3*^−*/*−^*Xiap*^−*/*−^ neutrophils were stimulated with TNF*α* (100 ng/ml) for indicated time points. Viability was assessed by flow cytometry; *n*=3, mean±S.E.M. (**c**) WT and *Xiap*^−*/*−^ neutrophils were primed with GM-CSF (1 ng/ml 30 min) followed by TNF*α* (100 ng/ml) treatment for 6 h. Lysates were assayed by immunoblot. Presented immunoblots are representative of two independent experiments. (**d**) Primed WT and *Xiap*^−*/*−^ neutrophils were stimulated with TNF*α* (100 ng/ml) for 0–45 min. Endogenous ubiquitylated proteins were isolated by TUBE1 and analyzed by immunoblot. Presented immunoblots are representative of at least two independent experiments. (**e**) *Xiap*^−*/*−^ neutrophils re-expressing WT or mutant XIAP, linked to an IRES-EGFP element, were investigated by immunoblot and primed as in (**f**) and stimulated with TNF*α* (100 ng/ml) for indicated time points. Presented immunoblots are representative of at least two independent experiments. Percentage of EGFP-positive cells is indicated (*n*=3). Viability was assessed by flow cytometry; *n*≥3, mean±S.E.M. All experiments were performed with *in vitro* differentiated neutrophils. *P<0.05, **P<0.01, ***P<0.005, ****P<0.001

Priming with the proinflammatory cytokine GM-CSF strongly protected primary and *in vitro* differentiated WT and *Xiap*^−*/*−^ neutrophils from spontaneous apoptosis (Figure 6a and Supplementary Figure S4a). Notably, whereas primed WT neutrophils were now refractory to high concentrations of TNF*α*, primed *Xiap*^−*/*−^ neutrophils remained sensitive to TNF*α*-induced killing (Figures 6a, b and Supplementary Figure S4a). Interestingly, GM-CSF priming increased expression levels of XIAP (Figure 6c and Supplementary Figure S1e). Furthermore, loss of XIAP resulted in increased ubiquitylation of RIPK1 and, to lesser extent, also of RIPK3 (Figure 6d). Whereas reconstitution of *Xiap*^−*/*−^ neutrophils with full-length XIAP restored resistance to TNF*α*-induced killing, expression of both BIR2 (XIAP L207P and XIAP C203Y) and RING domain (XIAP G466stop and XIAP P482R) mutants failed to rescue the cell death phenotype (Figures 6e and f). These data show that XIAP contributes to maintaining the viability of neutrophils under inflammatory conditions and indicate that both the BIR2 and the RING domain of XIAP are critical for the negative regulation of TNF*α*-induced cell death.

## Discussion

Besides its first identified role as direct caspase inhibitor, XIAP has recently been implicated in a RING domain-dependent positive regulation of NOD1/2 signaling in response to intracellular bacteria and in restriction of RIPK3-dependent cell death and inflammasome activation downstream of TNF-R1 and TLR4.^28, 31, 33, 35^ Most of these data are derived from macrophages or DCs. To date, little is known about the role of XIAP in innate signaling of neutrophils, which constitute a central first-line defense against invading pathogens, in particular bacteria and fungi. We therefore focused our study on LPS>TLR4- and TNF*α*>TNF-R1-mediated signaling and demonstrate a pivotal role of XIAP in regulating proinflammatory and cell death signaling in mouse neutrophils. In agreement with previous reports,^8, 49^ we show that neutrophils can secrete significant amounts of IL-1*β*. We show for the first time in neutrophils that, similar to macrophages and DCs,^27, 35^ loss of XIAP also promotes excessive IL-1*β* secretion in response to LPS (Figure 1). IL-1*β* secretion depended on RIPK3 and partly on caspase-1/-11, and based on the proteolytic activation of caspase-8 may additionally involve caspase-8-mediated processing of pro-IL-1*β*, as reported previously.^35^ In contrast to macrophages and DCs, however, neutrophils were shown to secrete IL-1*β* without undergoing pyroptosis.^49^ These data suggest that *Xiap*^−*/*−^ neutrophils may contribute to hyperinflammation and progression in certain pathologies seen in X-linked lymphoproliferative syndrome 2 patients. On the same line, mice lacking IAPs, including XIAP, may critically contribute to the immunopathogenesis observed in arthritis or during γ-herpesvirus infection.^27, 28^

Macrophages and certain tumor cells die in response to single treatment with Smac mimetics through NF-*κ*B- and autocrine TNF*α*>TNF-R1-dependent cell death.^48, 50, 51^ Intriguingly, Smac mimetics showed only weak killing potential *per se* on neutrophils and only when all three IAPs were targeted. Interestingly, this cell death was TNF*α*-dependent despite very low (or non-detectable) levels of soluble TNF*α* in the supernatants (Figure 2). This may suggest a role for membrane-bound TNF*α* in Smac mimetics-induced killing of neutrophils, which needs further investigations. Importantly, however, upon combined loss of all three IAPs, but not when XIAP was expressed, LPS stimulation resulted in increased TNF*α* secretion and markedly increased TNF*α*-dependent cell death. Our data demonstrate that this cell death occurs by classical apoptosis, and results in cleavage of the caspase-8 substrates RIPK1 and RIPK3 (Figures 3 and 4). Blockage of the enzymatic activity of RIPK1 by Nec.1 abolished caspase activation. This implicates that neutrophils undergo RIPK1-dependent apoptosis, similar to previous reports on other cell types.^48, 52^ Active RIPK1 is further critical for Smac mimetics-induced transcriptional induction of TNF*α* in macrophages.^48^ Similarly, Nec.1 blocked *Tnfα* production in LPS plus Smac mimetic-treated mouse neutrophils (Figure 4).

In agreement with the findings from LPS plus Smac mimetics treatment, direct addition of high concentrations of TNF*α* significantly increased cell death in both WT and *Xiap*^−*/*−^ neutrophils. It is worth discussing that unprimed *Xiap*^−*/*−^ neutrophils were not generally more sensitive than WT controls, arguing that a direct blocking of effector caspases by XIAP is inefficient to delay TNF-R1-mediated apoptosis in neutrophils (Figure 5). Targeting of cIAP1/-2, but not loss of XIAP, strongly sensitized neutrophils to lower concentrations of TNF*α*, indicating that cIAP1/-2 are important to prevent neutrophil cell death induced by low TNF*α*. Through its inhibitory action on activated effector caspases, XIAP has been shown to critically delay FasL/CD95L-induced apoptosis in type II cells, such as hepatocytes or pancreatic *β*-cells.^16^ Mouse neutrophils were reported to show type II characteristics in response to FasL/CD95L, but not in response to TNF*α*.^7, 8^ Importantly, we show that TNF*α* induces apoptosis in WT and *Xiap*^−*/*−^ neutrophils. However, in contrast to WT cells, this cell death could not be blocked in *Xiap*^−*/*−^ neutrophils using Q-VD-OPh unless RIPK1 or MLKL were also inhibited or RIPK3 was lost genetically. This implicates that in the absence of XIAP, caspase inhibition shifts TNF*α*-induced apoptosis to necroptosis. These findings are supported by our observation that MLKL translocated to the membrane under these conditions (Figure 5). It is somehow surprising that, despite high expression levels of RIPK1, RIPK3 and MLKL proteins at steady state (Figures 1 and 5), TNF*α*-treated WT neutrophils do not switch to necroptotic cell death under caspase-blocking conditions. Such a switch did, however, occur when XIAP was lost. Interestingly, loss of XIAP resulted in a further increase of RIPK1, RIPK3 and MLKL levels (Figure 5). Increased ubiquitylation of RIPK1 and RIPK3 was detected in TNF*α*-treated *Xiap*^−*/*−^ neutrophils, implying a role of XIAP in regulating stability and ubiquitylation status of those kinases (Figure 6). Our findings support the report by Yabal *et al.*^28^ in which XIAP was shown to limit necroptosis of DCs by regulating the stability of RIPK1 and RIPK3. Increased levels and ubiquitylation of RIPK1, RIPK3 and MLKL may, at least in part, explain the increased tendency of *Xiap*^−*/*−^ neutrophils to enter necroptosis. How XIAP regulates RIPK1 and RIPK3 is still under investigation. As loss of cIAP1/-2 causes a decrease in ubiquitylation of RIPK1,^25^ these findings point towards an indirect ubiquitylation of a yet unknown target protein by XIAP.

Further evidence for the involvement of XIAP in regulating TNF-R1 signaling were obtained upon GM-CSF priming, which mimics a proinflammatory environment and is known to increase the lifespan and activity of neutrophils.^3^ In agreement, primed WT neutrophils were protected from spontaneous apoptosis, which occurs through the mitochondrial apoptotic pathway.^9^ GM-CSF priming also conferred resistance to TNF*α*-induced cell death, even at high concentrations. In sharp contrast, *Xiap*^−*/*−^ neutrophils were protected from spontaneous apoptosis but remained sensitive to high TNF*α*. The finding that GM-CSF increased XIAP levels further supports a role of XIAP in survival of neutrophils. This phenotype could only be rescued upon re-expression of WT, but not BIR2 or RING domain mutants of XIAP in a *Xiap*^−*/*−^ genetic background. It is conceivable to hypothesize that the BIR2 domain may be needed for binding and the RING domain for ubiquitylation of a potential target protein, similar to reports on NOD signaling.^31^ These findings place XIAP as important negative regulator of TNF*α*-induced cell death under inflammatory conditions.

In summary, we have identified XIAP as an important downstream regulator of TNF*α*-induced cell death in mouse neutrophils. Loss of XIAP facilitates the switch from LPS>TNF*α*-induced apoptosis to RIPK3-dependent necroptosis and, importantly, also sensitizes GM-CSF-primed neutrophils to killing by TNF*α*. Furthermore, *Xiap*^−*/*−^ neutrophils secrete excessive amounts of IL-1*β* in response to LPS. Given the abundance of neutrophils at sites of infection and their essential role in early innate immune defense against invading pathogens, these findings may have direct implications to immunopathologies that are associated with loss-of-function mutations in XIAP, such as X-linked lymphoproliferative syndrome 2 (XLP2). The data further underline that unspecific targeting of IAP may cause unwanted side effects by compromising neutrophil viability.

## Materials and Methods

### Mice and reagents

C57BL/6 mice were maintained under pathogen-free conditions in IVC cages. *Xiap*^−*/*−^^53^ *and Ripk3*^−*/*−^ mice^54^ both backcrossed onto the C57BL/6 genetic background for at least eight generations, and *Xiap*^−*/*−^ *Ripk3*^−*/*−^ mice (from P Jost), have been described previously.^28^ Animal experiments were approved by the animal experimentation review board of the canton of Bern (BE31/11 and BE12/14).

RPMI-1640 AQmedia, 4-hydroxytamoxifen (4-OHT) and propidium iodide were purchased from Sigma-Aldrich Chemie GmbH (Buchs, Switzerland). Fetal calf serum (FCS; Sera Pro, ultra-low endotoxin) was purchased from Pan Biotech (Aidenbach, Germany). 2-Mercaptoethanol (2-ME) and penicillin/streptomycin were from Life Technologies (Carlsbad, CA, USA). CHO/SCF (mm) conditioned medium was used as a source of mouse stem cell factor and was produced as described previously.^45^ Recombinant FLAG-TNF*α* (mouse) and necrostatin-1 were purchased from Enzo LifeSciences AG (Lausen, Switzerland). AT-406 was from SelleckChem (Houston, TX, USA). Q-VD-OPh was purchased from SM Biochemicals (Anaheim, CA, USA). GW806742X was from Synkinase (Parkville, VIC, Australia). Recombinant mouse GM-CSF was from Peprotech (Rocky Hill, NJ, USA). Ultrapure LPS (*E. coli* K12) and ATP were from Invivogen (San Diego, CA, USA). Anti-TNF (Enbrel) was a kind gift from P Jost (Munich, Germany). IL-1 receptor antagonist (Anakinra) was a kind gift from P Villiger (Bern, Switzerland). Cp.A (GT12911) was produced by TetraLogic Pharmaceuticals (Malvern, PA, USA). Recombinant His^6^-tagged GFP-Annexin V was purified as described previously.^55^ Cherry-Annexin V was purified in-house. Agarose-TUBE1 (tandem-ubiquitin binding entity 1) was from LifeSensors (Malvern, PA, USA).

### *In vitro* differentiation of mouse neutrophils

Conditional Hoxb8 immortalized neutrophil/macrophage committed myeloid progenitors, termed SCF-^cond^Hoxb8 cells, were generated from bone marrow of WT, *Xiap*^−*/*−^, *Ripk3*^−*/*−^ and *Xiap*^−*/*−^*Ripk3*^−*/*−^ mice as described previously.^45, 56^ The cells were cultivated in RPMI-1640 AQmedia supplemented with 10% FCS, 1% penicillin/streptomycin, 5% SCF and 0.1 *μ*M 4-OHT. Mature neutrophils were obtained within 5 days upon removal of 4-OHT from the medium. To confirm differentiation, neutrophils were stained for the surface marker profile Gr-1(Ly-6C/Ly-6G)^hi^CD11b^+^CD117(c-kit)^neg^, using the following antibodies (all from BioLegend, San Diego, CA, USA): rat anti-Gr-1 (clone RB6-8C5), rat anti-CD11b (clone M1/70) and rat anti-CD117 (c-kit, clone 2B8). All experiments were performed with mature neutrophils.

### Generation of *Xiap*^−*/*−^ SCF-^cond^Hoxb8 neutrophils with reconstituted XIAP mutants

cDNAs of 3xHA-XIAP WT, the RING mutants, 3xHA-XIAP(G466stop) and 3xHA-XIAP(P482R), and the BIR2 mutants, 3xHA-XIAP(C203Y) and 3xHA-XIAP(L207P) (kind gift from M Gyrd-Hansen, Oxford, UK), were subcloned into the CAD-G-Whiz lentiviral expression vector, carrying an IRES-EGFP element,^57^ and subsequently transduced into *Xiap*^−*/*−^ SCF-^cond^Hoxb8 neutrophil progenitors. As a negative control, empty CAD-G-Whiz lentiviral expression vector was transduced into *Xiap*^−*/*−^ SCF-^cond^Hoxb8 neutrophil progenitors.

### Isolation of primary Gr-1^+^ neutrophils from bone marrow

Bone marrow cells were harvested from femoral bones. Gr-1^+^ cells were isolated by magnetic bead-based cell sorting according to the manufacturer's instructions (BD IMag; BD Biosciences, San Jose CA, USA) and using rat anti-Gr-1 (clone RB6-8C5) antibody from BioLegend. Cytospins of isolated neutrophils were stained with DiffQuik solution (Baxter, Deerfield IL, USA) and the morphology was checked under the light microscope. The purity was routinely above 95%. Isolated neutrophils were cultivated in RPMI-1640 AQmedia supplemented with 10% FCS, 1% penicillin/streptomycin and 50 *μ*M 2-ME.

### Assessment of cell death by flow cytometry

Neutrophils were stained with GFP-Annexin V or Cherry-Annexin V diluted in FACS buffer (150 mM NaCl, 4 mM KCl, 2.5 mM CaCl_2_, 1 mM MgSO_4_, 15 mM HEPES (pH 7.2), 2% FCS and 10 mM NaN_3_) for 20 min on ice in the dark. Cells were washed once with FACS buffer and resuspended in FACS buffer containing 2 *μ*g/ml of propidium iodide. Cells were analyzed by flow cytometry (FACSCalibur; BD Biosciences) and data were analyzed using WEASEL version 3.0.2. Cells negative for both GFP-Annexin V and propidium iodide were considered as viable.

### Gel electrophoresis and immunoblotting

Cells were lysed in IBC buffer (10 mM Tris-HCl, 1 mM EGTA, 200 mM sucrose, 2 mM MgCl_2_, 1% CHAPS, complemented with protease inhibitors (Roche Complete protease inhibitor cocktail plus 1 *μ*g/ml pepstatin), pH 7.6) where indicated or directly lysed in hot H8 buffer (20 mM Tris-HCl (pH 7.5), 2 mM EGTA, 2 mM EDTA, 1% SDS, supplemented with 50–100 mM DTT). Proteins from supernatants were precipitated as described previously.^58^ Proteins were denatured by adding 4 × Laemmli buffer (supplemented with 100 mM DTT) and boiling for 5 min. Proteins were separated on 7.5–15% denaturing SDS-PAGE gels and transferred to PVDF membrane (Immobilon-FL, 0.45 *μ*M; Merck Millipore, Zug, Switzerland) for detection. Membranes were probed overnight with the following primary antibodies: rabbit polyclonal anti-RIPK3 (ProSci, Poway, CA, USA; no. 2283); mouse anti-GAPDH (clone 6C5; Merck Millipore); mouse anti-RIPK1 (clone 610458), mouse anti-XIAP (clone 28/hILP/XIAP), mouse anti-PARP (clone C2-10) and mouse anti-actin (C4/actin) from BD BioSciences; rat anti-MLKL (clone 3H1, a kind gift from WS Alexander, Melbourne, VIC, Australia); rat anti-procaspase-8 (clone 1G12, a kind gift from L O'Reilly, Melbourne, VIC, Australia); rabbit anticleaved-caspase-8 (clone D5B2), rabbit polyclonal anti-procaspase-3 (no. 9662) and anticleaved-caspase-3 (no. 9661) from Cell Signaling (Danvers, MA, USA); rat anti-cIAP1 (clone 1E1-1-10, Enzo LifeSciences); mouse anti-porin (clone 89-173/016) from Calbiochem (Merck Millipore, Schaffhausen, Switzerland); mouse anti-tubulin (clone B-5-1-2) from Sigma (Buchs, Switzerland); rat anti-HA high affinity (clone 3F10) from Roche (Rotkreuz, Zug, Switzerland); mouse anti-NLRP3/NALP3 (clone Cryo-2, a kind gift from H-U Simon, Bern, Switzerland); rat anti-IL-1*β* (clone 166926; R&D, Bio-Techne, Minneapolis, MN, USA); rabbit anti-caspase-1 (clone EPR4321; Abcam, Cambridge, MA, USA). For TUBE1 purification assay and fractionation by phase separation experiments, secondary antibodies conjugated to horseradish peroxidase (Jackson ImmunoResearch Europe Ltd, Newmarket SFK, UK) were used and signals were obtained by enhanced chemiluminescence (Luminata Forte Western HRP substrate; Merck Millipore). For all immunoblots with total lysates, infrared dye-conjugated secondary antibodies (LI-COR Biosciences, Bad Homburg, Germany) were used. All immunoblots were analyzed with the Odyssey Fc Dual-Mode Imaging System using the ImageStudio software 3.1.4 (LI-COR Biosciences).

### TUBE1 purification

A total of 3–4 × 10^7^ neutrophils were stimulated as indicated and lysed in 3 ml IBC buffer (10 mM Tris-HCl, 1 mM EGTA, 200 mM sucrose, 2 mM MgCl_2_, 1% CHAPS, complemented with protease inhibitors (Roche Complete protease inhibitor cocktail plus 1 *μ*g/ml pepstatin), pH 7.6) for 30 min on ice. Lysates were cleared by centrifugation and ubiquitylated proteins were captured overnight at 4 °C on a rotating wheel using agarose-TUBE1, which displays a 10-fold higher affinity for K63-ubiquitylated proteins compared with K48-ubiquitylated proteins (LifeSensors, Malvern PA, USA). Beads were washed four times with IBC buffer and boiled for 5 min in 1 × Laemmli buffer containing 100 mM DTT.

### Cytokine ELISA

Mouse-specific IL-1*β*, IL-6 and TNF*α* ELISA Kits were from BioLegend and cytokine levels in cellular supernatants were measured according to the manufacturer's instructions.

### Detection of active caspase-3 and -7

A total of 2–4 × 10^5^ neutrophils were treated with 100 ng/ml of TNF*α* for indicated time points. Active caspase-3 and -7 were stained using CellEvent Caspase-3/7 Green Detection Reagent (Invitrogen, Thermo Fisher Scientific, Waltham, MA, USA) according to the manufacturer's instructions. Signals were acquired with a Zeiss AxioObserver.Z1 fluorescence microscope (Zeiss, Oberkochen, Germany) and by flow cytometry (FACSCalibur; BD BioSciences).

As a second approach, caspase-3 and -7 enzymatic activity was quantified by fluorometric assay using DEVD-AMC as a substrate (Bachem, Bubendorf, Switzerland). A total of 1 × 10^6^ neutrophils were lysed in 50 μl digitonin lysis buffer (20 mM HEPES (pH 7.4), 100 mM sucrose, 25 mM MgCl_2_, 100 mM KCl, protease inhibitor cocktail (Roche Complete protease inhibitor cocktail plus 1 *μ*g/ml pepstatin), 1 mM DTT, 0.025% digitonin). Assay buffer (0.1 M HEPES (pH 7.5), 10% sucrose, 0.1% CHAPS, 10 mM DTT) supplemented with 500 *μ*M DEVD-AMC (final concentration 50 *μ*M) was added to 10–25 *μ*g of protein lysate. Caspase activity was assessed kinetically for 1 h (1 min intervals) on a SpectraMax M2^e^ plate reader (Molecular Devices, Sunnyvale, CA, USA). Protein concentrations were measured by the BCA protein assay following the manufacturer's instructions (Thermo Fisher Scientific, Waltham, MA, USA).

### qPCR analysis

Total RNA was isolated from 5 × 10^6^ neutrophils using the SV Total RNA Isolation System (Promega, Wallisellen, Switzerland) according to the manufacturer's instructions. RNA was reverse transcribed using oligo d(T) primers and M-MLV reverse transcriptase (Promega) according to the manufacturer's instructions. Quantitative RT-PCR (qPCR) analysis was performed using HOT FIREPol EvaGreen qPCR Mix Plus from Solis Biodyne (Tartu, Estonia) on a Real-Time PCR machine (iQ5; Bio-Rad). Primer sequences were as follows: *mmTnfα* (TNF*α*, amplicon 128 bp) – Fw, 5′-ATGAGAAGTTCCCAAATGGC-3′ and Rev, 5′-CACTTGGTGGTTTGCTACGAC-3′ reference gene *mmHprt1* (HPRT, amplicon 124 bp) – Fw, 5′-TGGATACAGGCCAGACTTTGTT-3′ and Rev, 5′-CAGATTCAACTTGCGCTCATC-3′.

### Fractionation by phase separation

A total of 0.5–2 × 10^7^ neutrophils were treated as indicated and further lysed in 0.5–1 ml Triton X-114 lysis buffer (20 mM HEPES, pH 7.4, 150 mM NaCl, 2% Triton X-114, complemented with protease inhibitors (Roche Complete protease inhibitor cocktail plus 1 *μ*g/ml pepstatin)) for 30 min on ice (adapted from Wang *et al.*^59^). Lysates were cleared by centrifugation and further warmed at 30 °C for 3 min. Micelle-poor (Aq) and Micelle-rich (Det) phases were obtained by centrifugation (1500 × *g* for 5 min at RT). Aq phase was transferred to a new tube. Det phase was washed with a basal buffer (20 mM HEPES, pH 7.4, 150 mM NaCl) and finally diluted with the basal buffer to the same volume as the Aq phase. Proteins were denatured by adding 4 × Laemmli buffer (supplemented with 100 mM DTT) and boiling for 5 min.

### Statistical analysis

Data were analyzed using the Student's *t*-test. All values represent means±S.E.M. *P<0.05, **P<0.01, ***P<0.005 and ****P<0.001 were considered as statistically significant. Statistical analysis was performed using the Prism 6 software (GraphPad, La Jolla, CA, USA).

## Abbreviations

BIR: baculoviral IAP repeat
DC: dendritic cell
GM-CSF: granulocyte–macrophage colony-stimulating factor
IAP: inhibitor of apoptosis protein
IL-1*β*: interleukin-1*β*
IL-6: interleukin-6
LPS: lipopolysaccharide
Q-VD-OPh: quinoline-Val-Asp-difluorophenoxymethylketone
Smac: second mitochondria-derived activator of caspase
TLR: Toll-like receptor
TNF*α*: tumor necrosis factor-*α*
TNF-R1/2: tumor necrosis factor receptor-1/2
TUBE: tandem-ubiquitin binding entities
WT: wild-type
XIAP: X-linked IAP
