# Supplementary material for: Loss of XIAP facilitates switch to TNFα-induced necroptosis in mouse neutrophils
Source: Cell Death Dis. 2016 Oct 13;7(10):e2422–. doi: 10.1038/cddis.2016.311 (PMC5133978; doi:10.1038/cddis.2016.311)

Online Supplemental Information

Figure S1. Related to Figure 1.

(a) Primary mouse neutrophils were treated with LPS (100 ng/ml) for indicated time points. Viability was assessed by flow cytometry. n≥6, mean ± SEM. (b) Primary neutrophils were treated with LPS (100 ng/ml) for 24 h, and supernatants were analyzed for TNFα, IL-6 and IL-1β by ELISA. n≥3, mean ± SEM. (c) *In vitro* differentiated and (d) primary neutrophils, primed with GM-CSF (1 ng/ml for 30 min) or left unprimed, were stimulated with LPS (100 ng/ml) for 24 h and viability was assessed by flow cytometry. n≥3, mean ± SEM. (e) *In vitro* differentiated neutrophils were treated as indicated with GM-CSF (1 ng/ml) and LPS (100 ng/ml) for 6 h. Lysates were assayed by immunoblot. Presented immunoblots are representative of at least two independent experiments. (f) *In vitro* differentiated neutrophils were either primed with GM-CSF (1 ng/ml) or with LPS (100 ng/ml) for 30 min followed by treatment with LPS (100 ng/ml) or ATP (5 mM), respectively. Supernatants were collected and protein precipitated. Supernatant fractions and cell lysates were then assayed by immunoblot. Presented immunoblots are representative of at least two independent experiments. (g) *In vitro* differentiated WT and *Xiap*$^{-/-}$ neutrophils were pre-incubated with Q-VD-OPh (20 μM) or TNFα antagonist (10 μg/ml) for 30 min, respectively, and subsequently treated with LPS (100 ng/ml) for 3 h and 6 h. Lysates were assayed by immunoblot. Presented immunoblots are representative of at least two independent experiments.

Figure S2. Related to Figure 2.

(a) Primary neutrophils were pre-treated with AT-406 (1 μM) for 30 min and subsequently stimulated with LPS (100 ng/ml) for 24 h. TNFα and IL-1β in

supernatants were measured by ELISA. n≥3, mean ± SEM. (b) Primary neutrophils were pre-incubated either with AT-406 (1 μM) or Cp.A (500 nM) for 30 min and treated with LPS (100 ng/ml) for indicated time points. Viability was assessed by flow cytometry. n≥3, mean ± SEM. (c) Primary neutrophils were pre-treated with TNFα antagonist (10 μg/ml) for 30 min, further incubated with AT-406 (1 μM) or Cp.A (500 nM) and finally stimulated with LPS (100 ng/ml) for indicated time points. Viability was assessed by flow cytometry. n≥3, mean ± SEM. Same data sets of untreated control and SM ± LPS are shown in (b) and (c) to facilitate comparison.

Figure S3. Related to Figure 5.

(a) Primary neutrophils were pre-incubated with Q-VD (20 μM) and Nec.1 (20 μM) for 30 min as indicated and further treated with TNFα (100 ng/ml) for indicated time points. Viability was measured by flow cytometry. n≥4, mean ± SEM. (b) Primary neutrophils were treated with TNFα (100 ng/ml) for 16 h. Cells were stained for active caspase-3/-7 (green) using CellEvent Caspase-3/-7 Green Detection Reagent and PI (red). Presented images are representative of at least two independent experiments. Additionally, stained cells were analyzed by flow cytometry. (c) *In vitro* differentiated WT and *Xiap*$^{-/-}$ neutrophils were treated with indicated concentrations of TNFα. Viability was assessed by flow cytometry. n≥3, mean ± SEM. (d) *In vitro* differentiated WT and *Xiap*$^{-/-}$ neutrophils were pre-treated with either AT-406 (1 μM) or Compound A (500 nM) for 30 min and incubated with indicated concentrations of TNFα. Viability was assessed by flow cytometry. n≥3, mean ± SEM. Same data sets of untreated control from Fig. S1a (primary neutrophils) or Fig. 1a (*in vitro* differentiated neutrophils) are included.

Figure S4. Related to Figure 6.

(a) Primary neutrophils were primed with GM-CSF (1 ng/ml) for 30 min and then treated with TNFα (100 ng/ml) for indicated time points. Viability was measured by flow cytometry. n≥3, mean ± SEM.

Wicki et al. Supplementary Figure S1. Related to Figure 1

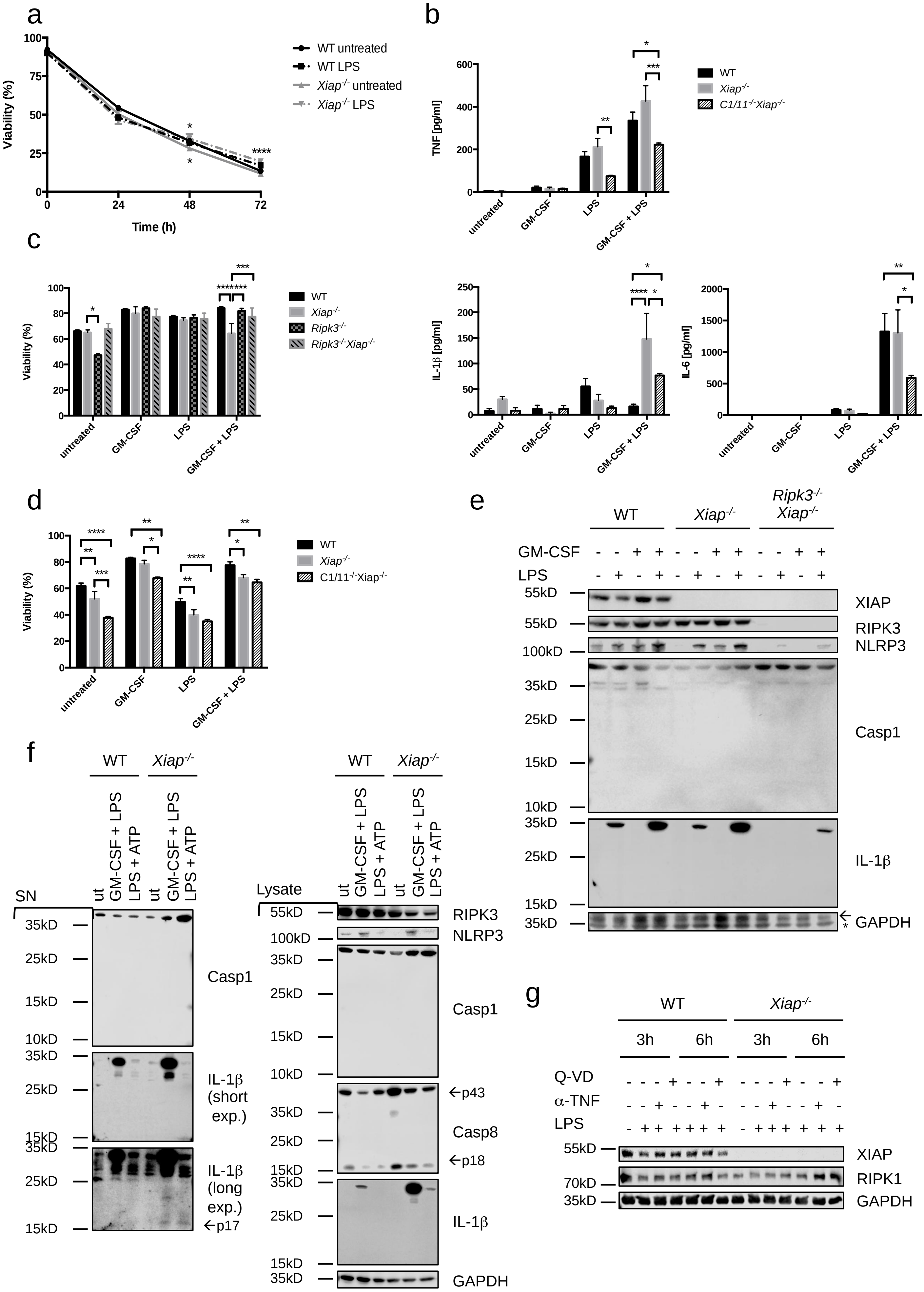

Wicki et al. Supplementary Figure S2. Related to Figure 2

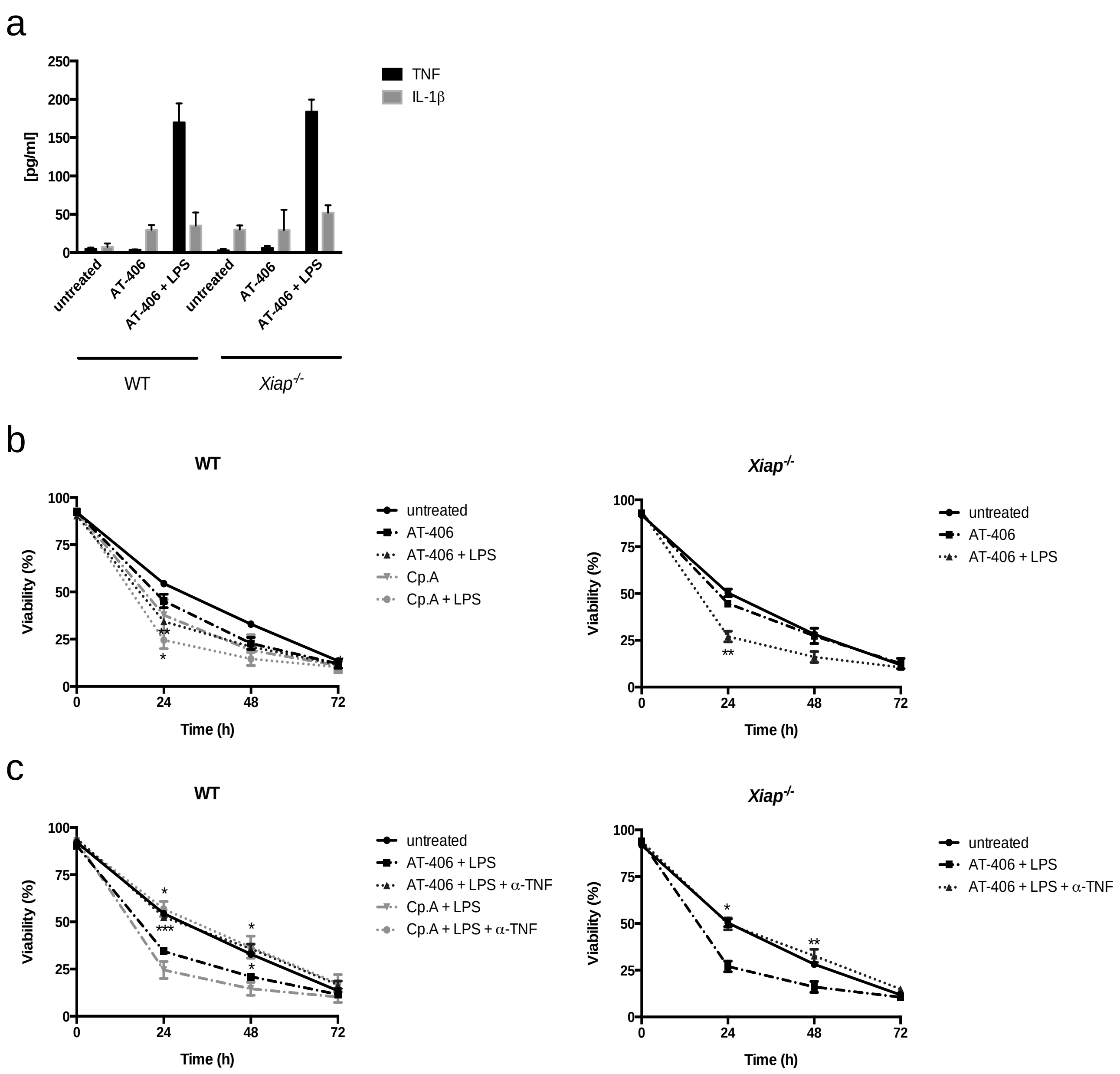

Wicki et al. Supplementary Figure S3. Related to Figure 5

a

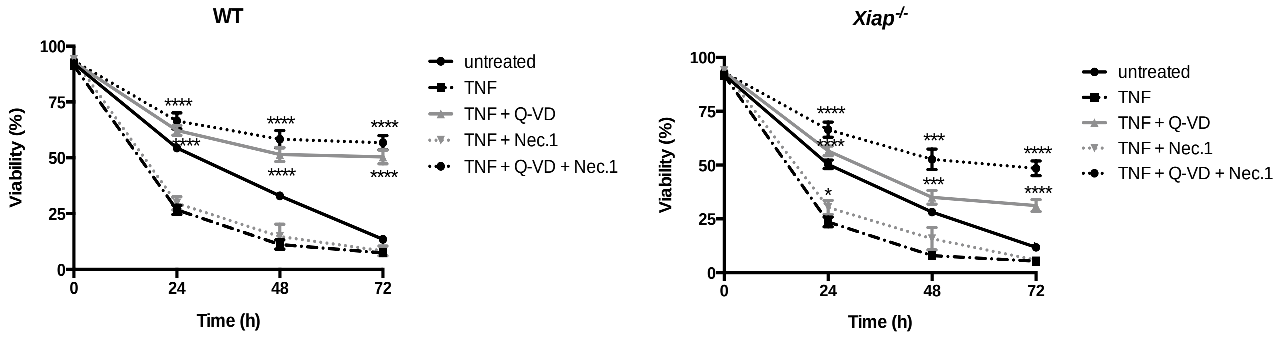

b

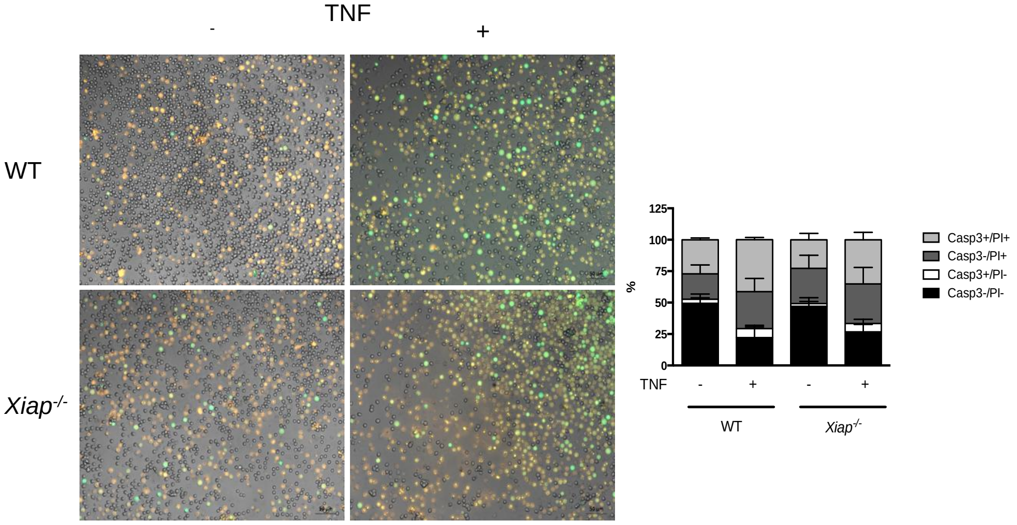

c

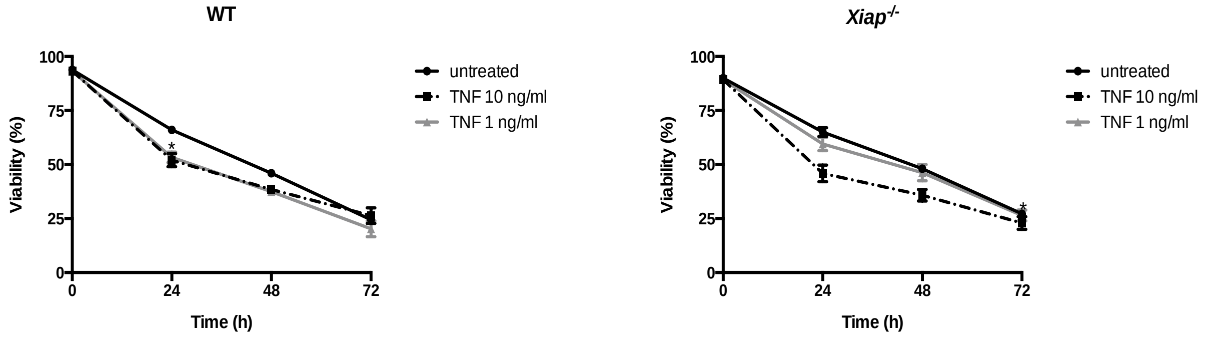

d

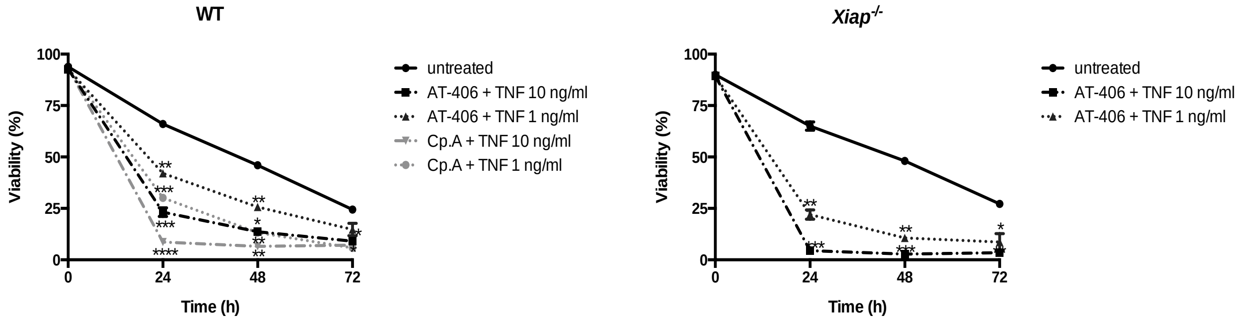

Wicki et al. Supplementary Figure S4. Related to Figure 6

a

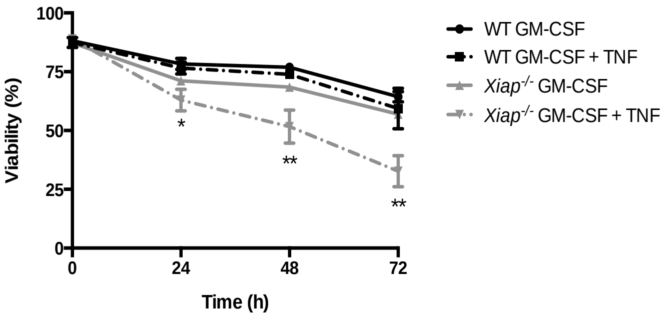

Supplement: Supplementary Information [file cddis2016311x1.pdf]
